# Surveillance efforts after mass drug administration to validate elimination of lymphatic filariasis as a public health problem in Vanuatu

**DOI:** 10.1186/s41182-017-0057-6

**Published:** 2017-06-16

**Authors:** Fasihah Taleo, George Taleo, Patricia M. Graves, Peter Wood, Sung Hye Kim, Masayo Ozaki, Hayley Joseph, Brian Chu, Alex Pavluck, Aya Yajima, Wayne Melrose, Kazuyo Ichimori, Corinne Capuano

**Affiliations:** 1 Government of Vanuatu, Vector Borne Disease Control Unit, Port Vila, Vanuatu; 2 College of Public Health, Medical and Veterinary Sciences, James Cook University, Cairns and Townsville, Queensland Australia; 3 WHO Office of the Representative for the South Pacific and Division of Pacific Technical Support, Suva, Fiji; 4 Liverpool School of Tropical Medicine, Liverpool, UK; 5 The Walter and Eliza Hall Institute of Medical Research, Parkville, Victoria Australia; 6 NTD Support Centre at the Task Force for Global Health, Atlanta, GA USA; 7 WHO Western Pacific Regional Office, Manila, Philippines; 8 WHO Headquarters, Geneva, Switzerland; 9 Nagasaki University, Nagasaki, Japan

## Abstract

**Background:**

Vanuatu was formerly highly endemic for lymphatic filariasis (LF), caused by *Wuchereria bancrofti* and transmitted by *Anopheles* mosquitoes. After a baseline survey showing 4.8% antigen prevalence in 1998, the country conducted nationwide (in one implementation unit) annual mass drug administration (MDA) with albendazole and diethylcarbamazine citrate from 2000 to 2004 and achieved prevalence of 0.2% by 2006 in a representative nationwide cluster survey among all age groups.

**Methods:**

Post MDA surveillance was conducted from 2006 to 2012. After MDA, the country was divided for surveillance into three evaluation units (EUs) formed by grouping provinces according to baseline prevalence: EU1: Torba, Sanma and Malampa; EU2: Penama; EU3: Shefa and Tafea. The study compiled all past data and information on surveys in Vanuatu from the country programme. This paper reviews the surveillance activities done after stopping MDA to validate the interruption of transmission and elimination of LF as a public health problem.

**Results:**

Post-MDA surveillance consisting of at least three transmission assessment surveys (TAS) in each of the three EUs was conducted between 2006 and 2012. Sentinel and spot check surveys identified a few villages with persistent high prevalence; all antigen positive cases in these sites were treated and additional targeted MDA conducted for 3 years in 13 villages in one area of concern. All three EUs passed all TAS in 2007, 2010 and 2012 respectively, with no positives found except in EU2 (Penama province) in 2012 when 2 children tested positive for circulating filariasis antigen. Assessment of the burden of chronic filariasis morbidity found 95 cases in 2003 and 32 remaining cases in 2007, all aged over 60 years.

**Conclusions:**

Vanuatu has achieved validation of elimination of LF as a public health problem. Post-validation surveillance is still recommended especially in formerly highly endemic areas.

## Background

Vanuatu is a Pacific Island nation and one of the 16 Pacific Island countries and territories included in the Pacific Programme to Eliminate Lymphatic Filariasis (PacELF) started in 1999 [1, 2]. PacELF is part of the Global Programme to Eliminate Lymphatic Filariasis (GPELF) which was launched in 2000 and aims to eliminate lymphatic filariasis (LF) as a public health problem by 2020 [3]. Elimination as a public health problem in this context includes interruption of transmission by mosquitoes and provision of services for those suffering from acute and chronic LF morbidity (acute attacks of lymphangitis, lymphoedema/elephantiasis and hydrocele).

Vanuatu is known to have been highly endemic for LF, based on a few surveys conducted prior to 1997, which have been reviewed in [4]. Some control efforts using mass drug administration (MDA) for up to 3 years in seven islands did not succeed in interrupting transmission [5]. At the start of the PacELF control programme in Vanuatu in 1999, the national LF antigen prevalence measured by immunochromatographic test (ICT) was estimated to be 4.8% in a baseline survey of 51 villages. Through intense efforts of annual MDA campaigns combining albendazole and diethylcarbamazine citrate (DEC) with vector control, antigen prevalence was reduced to 0.2% by 2006 as estimated in a survey of 90 household clusters. Programme implementation and MDA campaigns from 2000 until the final round of MDA in 2004 and the stop MDA survey in 2006 have been previously reported [4]. The current paper reviews the monitoring and post-MDA surveillance activities (including spot check site surveys after 2004) as evidence towards validation of elimination of LF as a public health problem according to current WHO validation process [6]. Available information on morbidity is also included in the present paper.

### The monitoring and surveillance framework

GPELF 2000 guidelines: Initially the Vanuatu LF programme followed the first (2000) GPELF guidelines [7]. These called for a mapping survey to assess LF endemicity in designated implementation units, testing of at least two sentinel sites and two spot check sites at baseline before the first round of MDA and during MDA, and, after achieving a microfilariae (Mf) prevalence rate of less than 1% in these sites, performing a lot quality assurance sampling (LQAS) survey of 3000 children aged 6–10 years, born after the initiation of effective MDA rounds. The LQAS survey was designed to determine whether further MDA rounds can be stopped using a cut-off of <0.1% circulating filariasis antigen (CFA) prevalence.

PacELF 2003 guidelines: Due to small population sizes of some Pacific Island countries and the lack of tests able to determine such a low level cut-off, the PacELF developed its own monitoring and evaluation (M&E) framework in 2003 and 2004 for assessing interruption of transmission [1]. This framework stipulated a community cluster survey of all ages, and MDA to be stopped if antigen prevalence was <1%. In the PacELF terminology, the baseline survey was A, the sentinel site surveys B, and the stop MDA survey was the C survey. The D survey was the LQAS survey among children as proposed by the GPELF.

Modified 2005 GPELF guidelines: In 2005, the GPELF published the revised M&E guidelines [8] which recommended increased numbers of sentinel and spot check surveys be conducted before the fifth MDA to determine whether the Mf prevalence in all of the sampled sites was <1%. To determine whether MDA should be stopped, the LQAS survey of 3000 children was retained, but the recommended age group was changed to school entrants. However, Vanuatu was already in the process of conducting a C survey in all age groups in 2005 [4], rather than following the 2005 GPELF guidelines.

Modified PacELF 2008 guidelines: In 2007, the PacELF proposed a new surveillance framework that was modified in 2008 [9] for the Pacific countries, including Vanuatu. These guidelines retained the community-based cluster C survey in all ages for the stopping MDA decision with a threshold of 1% antigen prevalence but modified the D survey to a child transmission survey (CTS). The CTS had a target sample size of all children aged 5 in a country (with the exception of PNG), to detect antigen positive children, either in school or community-based surveys. An additional action component included was the tracing of contacts of any cases who were positive for CFA (by ICT) or Mf, by testing surrounding residents within 200 m or the nearest 24 houses to the index child’s place of residence. Vanuatu adopted this approach and conducted a nationwide CTS in 2007. The 2008 PacELF framework used in Vanuatu was implemented to some extent in other Pacific countries.

GPELF 2011 guidelines: The modified PacELF framework used until 2008 was then superseded by the new WHO-recommended protocol for transmission assessment surveys (TAS) [10] which was the procedure followed by Vanuatu for subsequent post-MDA surveys after 2010. PacELF followed these guidelines for TAS after 2008.

## Methods

### LF programme timeline

Table 1 shows the summarised timeline of activities between 1997 and 2012, including specific timing of post MDA surveillance and follow-up activities described in this paper.

**Table 1:** Surveillance activities in Vanuatu, 1997–2012

Year	M&E activity	Detail and location by province or evaluation unit (EU)
1997–1998	Mapping survey: baseline A survey	Nationwide, 51 villages
2000	The first year of MDA	Nationwide
2002	Sentinel site B surveys	Torba, Sanma, Malampa, Penama, 2 villages each
	Spot checks	1 village Malampa, 1 village Penama, 2 hospitals
2003	Spot checks	4 hospitals
	Morbidity assessment	Nationwide
2004	The last year of MDA	Nationwide
	Spot checks	15 villages, North Ambrym, Malampa
2005 To 2006	Stop MDA survey C survey/TAS 1 (all ages)	Nationwide in 30 villages per EU EU1 (Torba, Sanma, Malampa); EU2 (Penama); EU3 (Shefa and Tafea)
	Sentinel sites	Torba, Sanma, Penama (2 villages each).
	Spot checks	6 villages of Penama (EU2)
2007	Transmission assessment survey D survey/CTS/TAS 2	Community TAS 2 in 6–7 year olds EU1 (Torba, Sanma, Malampa); EU2 (Penama); EU3 (Shefa and Tafea)
	Morbidity assessment	Malampa and parts of Penama, Sanma, Shefa, Tafea
2008	Spot checks	5 villages of South Pentecost and West Ambae (Penama, EU2)
	Targeted MDA^a^ and spot checks	Targeted follow-up MDA round 1: 13 villages of North Ambrym, Malampa province (in EU1) Spot checks followed MDA
2009	Targeted MDA	Targeted follow-up MDA round 2: North Ambrym, Malampa province (in EU1)
2010	Targeted MDA	Targeted follow-up MDA round 3: North Ambrym, Malampa province (in EU1)
	Transmission assessment survey TAS 3	Community TAS 3 in EU1 and 2; Children tested in 2 spot-check villages in EU3
2011	Spot checks and sentinel sites	Vila and Santo Hospitals USP students Sentinel sites EU1, EU2, EU3 (2 villages each—test and treat of former positives only)
2012	Transmission assessment survey TAS 3 continued	Community TAS 3 in EU3
	TAS 4	Community TAS 4 in EU2
	Dossier preparation	Preparation of elimination dossier started

^a^In targeted MDA, treatment without prior testing is offered to all inhabitants >2 yrs of age in selected villages thought to have persistent high prevalence, such as villages of North Ambrym identified in 2004. See [4] for details

Following the baseline survey in 1997 and 1998, annual MDA rounds were conducted for five consecutive years nationwide during 2000–2004, with reported national coverage rates of 83, 84, 84, 87 and 85% respectively [1, 4, 11]. Sentinel site B surveys were started in eight villages in 2002 [12]. Spot-check site surveys were also initiated in several villages and hospitals between 2002 and 2004 during the MDA period [4]. The last MDA was conducted in 2004, and the C survey was done in 2005–2006 (together with sentinel sites and additional spot-check site surveys), and CTS or TAS surveys in 2007, 2010 and 2012.

## Results

### Sentinel and spot-check site surveys and targeted MDAs

**Fig. 1:**
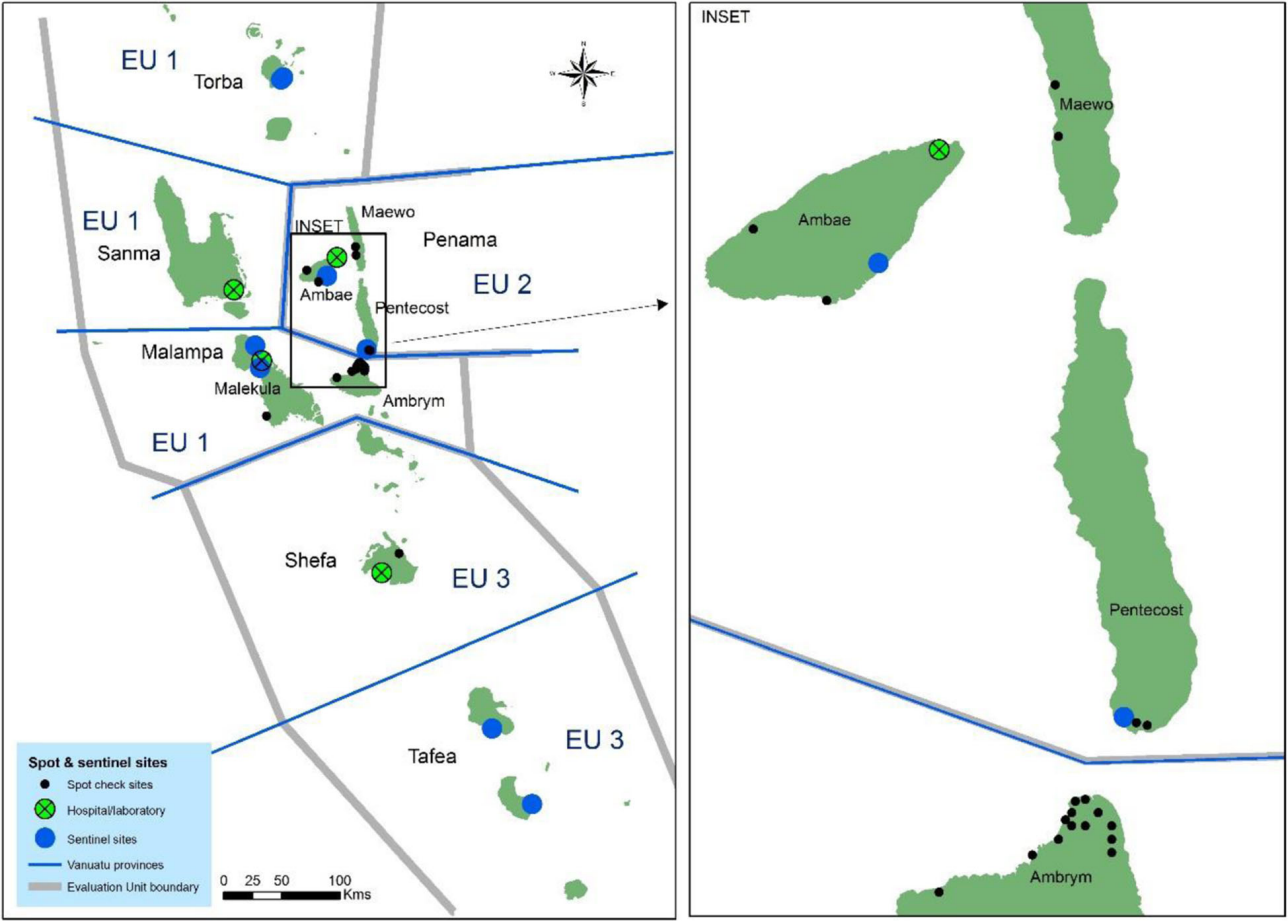
Division of Evaluation Units (EUs), and locations of sentinel and spot check sites and hospitals. Locations of the sites surveyed between 2002 and 2012 are shown (excluding the C survey 90 villages). The inset shows detail of North Ambrym (Malampa province, EU1) and islands of Penama province (EU2)

As reported previously and summarised in Table 2 [4], there were six sentinel sites (two each in Torba, Sanma and Penama provinces) that were surveyed in 1997/1998, 2002 and 2006. Two additional sentinel sites in Malampa province were surveyed in 1997/1998 and 2002 only. The names of these eight sentinel site villages are shown in italics in Table 2. All sentinel sites showed a decline in CFA prevalence over time, and five of the six sites had reached 0% CFA prevalence by 2006. The remaining site with persistent positives was Wanur in South Pentecost, Penama province. The locations of the sentinel sites are indicated in Fig. 1.

**Table 2:** Results of sentinel and spot check site surveys by village, 1998–2011

EU	Province	Island	Village^a^	CFA prevalence by ICT (number tested)					
				1998	2002	2004	2005/2006	2008	2010
1	Torba	Vanua Lava	*Sola*	9.7% (31)	1.2% (165)		0.0% (154)		
			*Mosina*	10.7% (28)	4.0% (76)		0.0% (80)		
			Vetimbuso				6.0% (60)		
	Malampa	Ambrym	North Ambrym 13–15 villages			19.1% (551)		2.3% (1368)^b^	
			*Unmet*	52.0% (100)	20.0% (208)				
		Malekula	Lingarak		0.0% (218)				
			*Orap*	4.0% (100)	1.3% (224)				
2	Penama	Ambae	*Sakau*	45.8% (48)	27.2% (92)		0.0% (61)		
			Redcliffe		39.5% (129)		0.0% (132)		
			Nanako					0.0% (123)	
		Maewo	Baitora	77.8% (72)			4.8% (84)		
			Nasawa	20.7% (121)			4.1% (121)		
		Pentecost	Beimateli/ Londar	43.9% (155)			6.1% (213)		
			Point Kross					3.9% (333)^c^	
			*Wanur*	35.0% (60)	8.6% (58)		2.3% (88)		
3	Shefa	Efate	Ebule						0.0% (91) ^d^
	Tafea	Tanna	*Port Resolution*	7.1% (98)	1.3% (300)		0.0% (135)		0.0% (50) ^d^
		Erromango	*South River*	13.9% (65)	6.2% (65)		0.0% (100)		

^a^Original sentinel site villages are shown in italics. In sentinel sites in 2002, only persons aged over 10 years were tested

^b^Also tested: 3–6 year olds in 13 North Ambrym villages, 2008: 0.0% (188); only combined data available

^c^Also tested: 3–6 year olds in 4 Pentecost villages (Barmateli/Londar, Namaram, Point Kross, and Wanur), 2008: 0.0% (86); only combined data available

^d^These surveys done in 6–7 year old children only, as part of TAS 3

Some additional villages were sampled as spot-check sites, some of them more than once, from 2002 onwards. These surveys are also compiled in Table 2, with locations of the sites indicated in Fig. 1. The surveys tested all age groups, with additional tests done on 3–6 year olds or 6–7 year olds in certain villages, as shown as footnotes to the table.

It can be noted from Fig. 1 that North Ambrym (1999 census population 3899) and South Pentecost (2000 estimated population 2222) are located close to each other, despite being in different provinces and evaluation units (EUs). North Ambrym was identified as an area of concern due to remaining high prevalence in 2004 from spot-check site surveys, and Maewo and South Pentecost in 2005–2006 through spot-check sites done in conjunction with the C survey. Baitora and Nasawa villages (in Maewo) and Beimateli/Londar village (in south Pentecost) as spot-check sites all had >4% CFA prevalence in 2006. There was also 6% CFA prevalence (*N* = 60) in one village (Vetimboso) surveyed in Torba province in 2006.

In response to the spot-check site survey results in North Ambrym in 2004, the programme decided to implement three additional rounds of targeted MDA (offering treatment to all village residents) in this area in 2008, 2009 and 2010 with treatment coverage of 76, 78 and 79%. Spot checks in 2008 covered 13 villages (Konkon, Fantongtong, Ranvetlam, Ranon, Fona, Olal, Makam, Wilit, Noha, Nimbul, Nobul, Fanla and Fanrereo) after the first targeted MDA, and the results showed that CFA prevalence had dropped from 19.1% in 2004 to 2.3% in 2008 in all age groups (Table 2). The survey of 188 children aged 3–6 years in the same area found no CFA positives.

In South Pentecost in 2008, a spot-check survey found 3.9% CFA prevalence in a newly sampled site, Point Kross. Additional testing of 3 to 6 year olds only was done in 4 villages in South Pentecost in 2008: Point Kross, Londar/Baemateli, Namaram and the former sentinel site Wanur (Table 2), with no CFA positives detected. Another spot-check site survey (Nanako village in Ambae) in the same EU showed 0% positive out of 123 tested.

In summary, there were 46 CFA positives detected (32 in North Ambrym and 14 in South Pentecost) whose ages ranged from 17 to 73 years old in 2008. Eight of the CFA-positive cases in South Pentecost had been positive in the baseline survey in 1997/1998. The others had not been tested until 2005 or 2008. There were no Mf positives found among those who tested positive for CFA in any sites surveyed; all CFA positives were treated directly after Mf blood slides were collected. Unlike in North Ambrym, no further MDA was conducted in Pentecost or Maewo, where villages with persisting prevalence >1% were discovered in 2005–2006, since sentinel and spot-check sites had all been offered testing and treatment if positive; in addition, Penama province (EU2) received an extra TAS in 2012 (see below).

In 2011, ICT testing (*N* = 1100) was also conducted at 2 main hospitals (in Santo and Port Vila) on persons presenting for malaria examinations, and zero CFA positives were found. As for migrant screening, the programme tested 101 students who came to study at the University of South Pacific in Port Vila from other Pacific Island countries and found zero CFA positives. The formerly positive persons in all 8 sentinel sites from 2002 to 2008 were also followed up and retreated if still positive in 2011. There were 2 positive persons found out of 102 tested in all sites in 2011, and they were both resident in Sakau village in Penama (EU2) where 23 of the formerly positive people were tested.

### Transmission assessment surveys

The C survey of 2005/2006 is now referred to as TAS 1, although it was done in all ages rather than children aged 6–7 years as recommended by the transmission assessment surveys (TAS) protocol [10]. Results were presented in [4].

For TAS 2, conducted in November and December 2007, Vanuatu piloted the 2008 revised PacELF guidelines; this survey is also referred to as the D survey or child transmission survey (CTS) in the original [1] and revised [9] PacELF framework respectively. The CTS/TAS 2 in 2007 tested 71.9% of the estimated total number of eligible 6- and 7-year-old children (born between 1 November 2000 to 31 December 2001) in three EUs comprising all six provinces as grouped for the C survey/TAS 1 (estimated *N* = 6605) through a community-based approach (Table 3). There were 25 teams in total and 2 to 6 teams were assigned per province. The teams covered 86.2% of the target number of children in EU1, 52.3% in EU2 and 71.8% in EU3. No CFA positives were found among the 4752 children tested, aged 6–7 years, giving upper 95% confidence intervals for CFA prevalence of 0.2% in EU1, 1.0% in EU2 and 0.3% in EU3 (Table 3). The survey sample builder software [10] was not used for these surveys, but no positives were detected so the results were below any threshold that would have been generated. Thus, in TAS 2, all three EUs passed.

**Table 3:** Results of TAS 2 in all EUs, 2007

Evaluation unit	Province	Province 6–7 year old pop^a^	% of 6–7 year pop tested	*N* tested ICT	*N* ICT positive	% ICT positive	Upper 95% exact binomial CI
1	Torba	355	77.2%	274	0	0.0%	
	Sanma	1305	73.6%	960	0	0.0%	
	Malampa	1128	86.2%	972	0	0.0%	
	Total EU1	*2788*	*79.1%*	*2206*	*0*	*0.0%*	*0.2%*
2	Penama	966	52.3%	505	0	0.0%	
	Total EU2	*966*	*52.3%*	*505*	*0*	*0.0%*	*1.0%*
3	Shefa	1711	86.1%	1473	0	0.0%	
	Tafea	1140	49.8%	568	0	0.0%	
	Total EU3	*2851*	*71.6%*	*2041*	*0*	*0.0%*	*0.3%*
Vanuatu total		*6605*	*71.9%*	*4752*	*0*	*0.0%*	*0.1%*

^a^Projected from 1999 census

TAS 3 was conducted in 2010 and 2012 under GPELF 2011 guidelines [10]. EU1 and EU2 were surveyed in 2010 using a school-based approach, testing all first graders in the respective EUs. TAS 3 in EU3 initially tested only children in one sentinel site (Port Resolution, Tafea province) and one spot-check village (Ebule, Shefa province) in 2010. Completion of the TAS 3 in EU3 in all other school-aged children was done in 2012. Over both years combined, 63.2% (77.1% in EU1, 90.1% in EU2 and 40.8% in EU3) of all 6–7-year-old children in the three EUs were tested with ICT (estimated total population *N* = 7086) (Table 4) and no CFA positives were found among the 4480 children tested. Despite the low sampling proportion in EU3, all three EUs passed the TAS 3 (Table 4), as the results were below the critical cut-offs (first integer <0.02*N*, where *N* is the estimated population in each EU). The TAS 3 in EU2 was conducted with support from the Task Force for Global Health, and the results have been published in summary form [13].

**Table 4:** Results of TAS 3 in all EUs, 2010 and 2012

Evaluation unit	Province	Province 6–7 year old pop^a^	% of 6–7 year pop tested	*N* tested ICT	*N* ICT pos	% ICT pos	Upper 95% exact binomial CI
1	Torba (2010)	380	19.2%	73	0	0.0%	
	Sanma (2010)	1397	113.6%	1587	0	0.0%	
	Malampa (2010)	1207	53.3%	643	0	0.0%	
	Total EU1	*2984*	*77.2%*	*2303*	*0*	*0.0%*	*0.1%*
2	Penama (2010)	1034	89.9%	930	0	0.0%	
	Total EU2	*1034*	*90.1%*	*930*	*0*	*0.0%*	*0.4%*
3	Shefa (2010)^b^ (2012)	1831	5.0% 35.7%	91 653	0 0	0.0% 0.0%	
	Tafea (2010)^b^ (2012)	1,220	4.1% 37.1%	50 453	0 0	0.0% 0.0%	
	Total EU3	*3051*	*40.8%*	*1247*	*0*	*0%*	*0.2%*
Vanuatu total		*7086*	*63.2%*	*4480*	*0*	*0%*	

^a^Projected from 1999 census

^b^TAS 3 in EU3 in 2010 was done in one village site in each province only (Ebule in Shefa province and Port Resolution in Tafea province)

A TAS 4 was performed only in EU2 (Penama province) in 2012 (Table 5). The estimated total population of 6–7-year-old children was 1034 in the EU. Two ICT positive children were found out of 933 tested. The critical cut-off was 20 and therefore EU2 passed TAS 4 [10]. The results of the TAS 4 have been published in summary form [13].

**Table 5:** Results of TAS 4 in EU2, 2012

Evaluation unit	Province	Province 6–7 year old pop^a^	% of 6–7 year pop tested	*N* tested ICT	*N* ICT pos	% ICT pos	Upper 95% exact binomial CI
2	Penama (2012)	1034	90.2%	933	2^b^	0.2%	
	Total EU2	*1034*	*90.2%*	*933*	*2*	*0.2%*	*0.8%*

^a^Projected from 1999 census

^b^Both were Mf negative

### Morbidity burden estimates

**Fig. 2:**
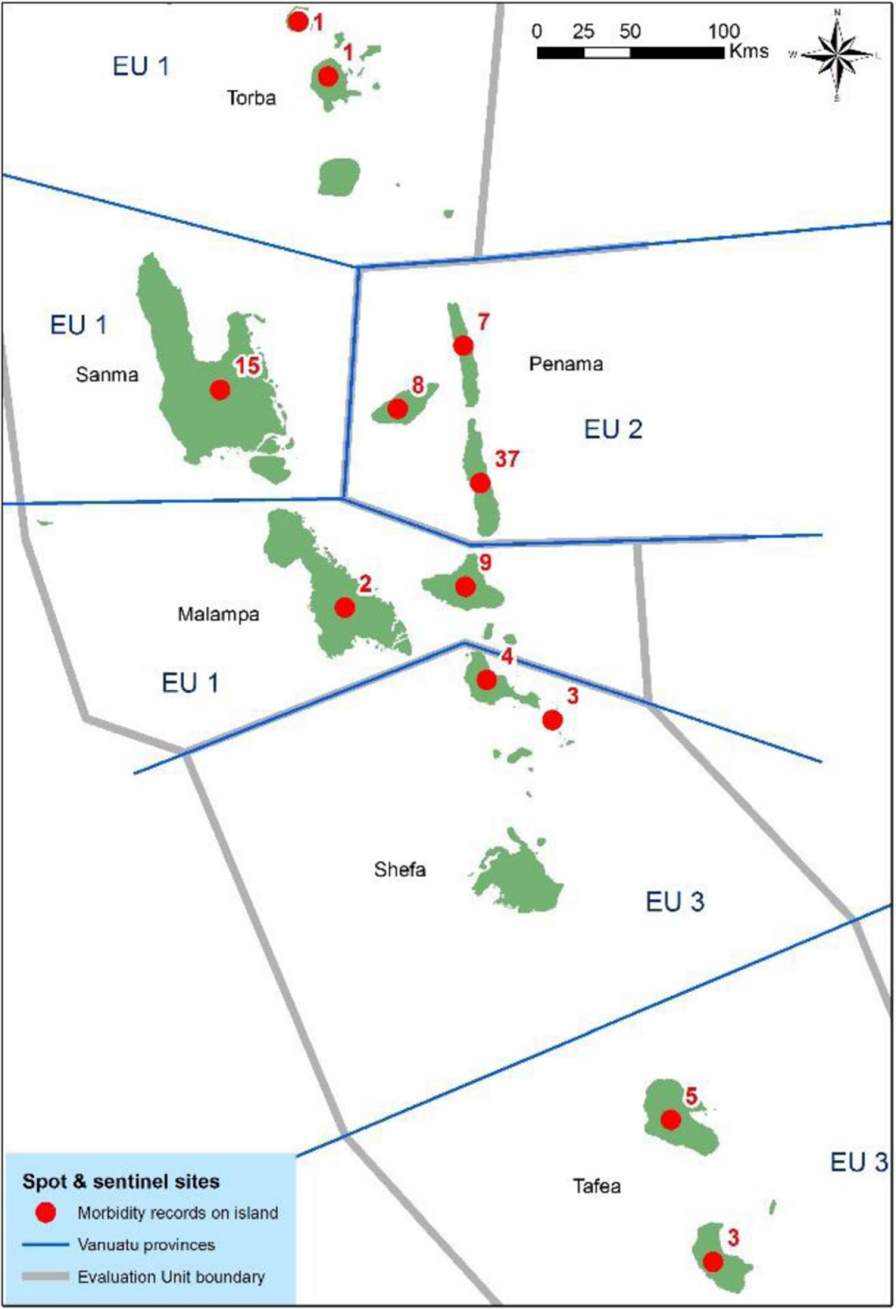
Location and number of morbidity cases identified in 2003

In 2003, the LF programme attempted to identify all persons with LF morbidity in the country by enlisting health staff to investigate during the MDA round. A morbidity survey form was inserted into the 2003 MDA registration books, and nurses were instructed to record any morbidity patients in their area on the forms which were submitted back to the national programme after the MDA. The resulting estimates are shown in Table 6, and the numbers and location by island in Fig. 2. A total of 95 cases were found in 2003, with two thirds being in males; about one third of cases were hydroceles or mixed hydroceles, or mixed limb and breast-related morbidities.

**Table 6:** Morbidity data, 2003 and 2007

Province	Island	2003									2007		
		No cases	M	F	Body part affected						No cases	M	F
					Leg	Arm	Hydrocele	Breast	Mixed	Unknown			
TORBA	Ureparapara	1	1	0			1						
	Vanualava	1	1	0	1								
	*Total TORBA*	*2*	*2*	*0*	*1*		*1*						
SANMA	Santo	15	8	7	8	1	2		4				
	*Total SANMA*	*15*	*8*	*7*	*8*	*1*	*2*	*0*	*4*	*0*	*9*	*3*	*2*
MALAMPA	Ambrym	9	7	2	6		2	1			5	5	
	Malekula	2	2			1	1				2	2	
	Paama										1		1
	*Total MALAMPA*	*11*	*9*	*2*	*6*	*1*	*3*	*1*	*0*	*0*	*8*	*7*	*1*
PENAMA	Ambae	8	6	2	5		2	1					
	Maewo	7	5	2	6		1						
	Pentecost	37	18	19	26	2	6	1	2		8	5	3
	*Total PENAMA*	*52*	*29*	*23*	*37*	*2*	*9*	*2*	*2*	*0*	*8*	*5*	*3*
SHEFA	Epi	4	2	2	1	1	1		1		6	2	4
	Tongoa	3	2	1	1		2						
	*Total SHEFA*	*7*	*4*	*3*	*2*	*1*	*3*	*0*	*1*	*0*	*6*	*2*	*4*
TAFEA	Tanna	3	3	0	1		1			1	1	1	
	Erromango	5	5	0	1		4						
	*Total TAFEA*	*8*	*8*	*0*	*2*		*5*	*0*	*0*	*1*	*1*	*1*	*0*
VANUATU TOTAL		95	60	35	56	5	23	3	7	1	32	18	10

In 2007, during the CTS/TAS 2, an attempt was made to determine an updated estimate and case register of LF morbidity cases with local nurses. The teams carried with them sufficient morbidity kits for all the previously identified cases (care charts, basins, towels and bars of soap) after the team members were trained in morbidity management. At this second round of the assessment, only 32 people with morbidity were followed up (18 males and 10 females, 4 in Sanma province with unknown gender) compared to 95 in 2003 (Table 6); however, it was reported that some of the survey teams did not have time to do thorough morbidity assessment. The cases recorded in 2007 were all above 60 years of age, and again, the majority had limb-related morbidity rather than hydroceles. Decrease in numbers of cases between 2003 and 2007 was seen in all provinces, with the biggest decrease being on Pentecost Island in Penama province (EU2).

### Availability of hydrocele surgery and numbers performed

During the period 1998 to 2006, hydrocele surgery was available in Port Vila hospital, and an estimated 10–15 surgeries were performed during that period on hydroceles of stages 2 to 4 [14]. After 2006, this surgery was not available in Vanuatu until a visit by the external surgeon in November 2015. There were at least 23 cases of hydroceles with surgical backlog, of which eight cases were presented for examination. However, out of these 8 patients selected by referring physicians with a diagnosis of hydrocele, all 8 were actually confirmed as inguinal hernia. All were of big size, most of them were non reducible and none was strangled. While no hydrocelectomy was performed at that time, the fact that all referred cases were hernia indicates the need for further training in the differential diagnosis of hydroceles and poses the question of the real number of remaining patients affected by “hydroceles” out of the remaining 15 (23 minus 8) from Table 4 as part of post-validation activities of LF morbidity management.

## Discussion and Conclusions

Vanuatu’s national CFA prevalence by ICT before MDA in 1998 was 4.8% (*N* = 4362) and after MDA in 2005 was 0.2% (*N* = 7580) [4]. When considered by EU, which is a combination of provinces used to design sampling frames for the C survey/TAS 1 in 2005/2006, the CFA prevalence immediately after MDA was 0.1% in EU1 comprising Torba, Sanma and Malampa (*N* = 2790 tested), 0.2% in EU2 Penama (*N* = 2592 tested) and 0% in EU3 comprising Shefa and Tafea provinces (*N* = 2198 tested).

This paper reports the details about monitoring activities during the MDA and the post MDA surveillance period. In the TAS 2 and 3, all three EUs passed according to the WHO criteria. Due to concerns about persistence of high prevalence of 3.9% in at least one village identified in 2008, an additional TAS 4 was conducted in Penama province only (EU2) in 2012. In this TAS 4, EU2 passed; although there were 2 CFA positive children discovered, the number of positives was well below the TAS threshold. Further post validation surveillance, including antibody tests in children, would be advisable especially in South Pentecost to ensure that transmission is not persisting any longer.

The dossier summarising all the information about the LF programme in Vanuatu was prepared in 2012 and submitted to WHO in 2013. It was reviewed during the 13th Meeting of the Western Pacific Regional Programme Review Group (RPRG) on neglected tropical diseases (NTDs) in July 2013, which suggested a few modifications. The dossier was updated accordingly and forwarded to WHO headquarters for necessary actions in October 2014. The WHO Strategic and Technical Advisory Group on NTDs endorsed the official process to validate elimination of LF as a public health problem in April 2015. As per the new process, elimination of LF as a public health problem is to be validated by the regional reviewing authority convened by the relevant WHO Regional Office and acknowledged by the WHO Director General. Accordingly, the Regional Dossier Review Group was convened by the WHO WPRO, which reviewed the dossier of Vanuatu and recommended validation of the claim for Vanuatu. An official acknowledgement of accomplishment of LF elimination as a public health problem in Vanuatu was given by the WHO Director General and the WHO WPRO Regional Director during the 67th session of the Regional Committee Meeting held in Manila in October 2016.

## Abbreviations

A survey: PacELF baseline mapping survey
B survey: PacELF equivalent of sentinel site survey
C survey: PacELF stop MDA survey done in all ages. Revised version would be TAS 1 but that is done in children only
CFA: Circulating filariasis antigen
CTS: Child transmission survey
D survey: PacELF’s equivalent to child transmission survey and TAS but with different target threshold and sample size
DEC: Diethylcarbamazine citrate
EU: Evaluation unit
GPELF: Global Programme to Eliminate Lymphatic Filariasis
ICT: Immunochromatographic test
LF: Lymphatic filariasis
LQAS: Lot quality assurance sampling
MDA: Mass drug administration
Mf: Microfilariae
NTD: Neglected tropical diseases
PacELF: Pacific Programme to Eliminate LF
RPRG: Regional Program Review Group
TAS: Transmission assessment survey
USP: University of the South Pacific
VBDCU: Vector Borne Disease Control Unit
WHO: World Health Organization
WPRO: WHO Western Pacific Regional Office
